# Adiponectin, biomarkers of inflammation and changes in cardiac autonomic function: Whitehall II study

**DOI:** 10.1186/s12933-017-0634-3

**Published:** 2017-12-01

**Authors:** Christian Stevns Hansen, Dorte Vistisen, Marit Eika Jørgensen, Daniel R. Witte, Eric J. Brunner, Adam G. Tabák, Mika Kivimäki, Michael Roden, Marek Malik, Christian Herder

**Affiliations:** 1 Steno Diabetes Center Copenhagen, Niels Steensens Vej 2-4, 2820 Gentofte, Denmark; 2 Department of Public Health, Aarhus University, Aarhus, Denmark; 3 Danish Diabetes Academy, Odense, Denmark; 4 Department of Epidemiology and Public Health, University College London, London, UK; 5 First Department of Medicine, Faculty of Medicine, Semmelweis University, Budapest, Hungary; 6 Institute for Clinical Diabetology, German Diabetes Center, Leibniz Center for Diabetes Research at Heinrich Heine University Düsseldorf, Düsseldorf, Germany; 7 German Center for Diabetes Research (DZD), München-Neuherberg, Germany; 8 Division of Endocrinology and Diabetology, Medical Faculty, Heinrich Heine University Düsseldorf, Düsseldorf, Germany; 9 National Heart and Lung Institute, Imperial College, London, UK

**Keywords:** Cardiovascular disease, Cardiovascular autonomic neuropathy, Heart rate, Inflammation, Adipokine, Prediction

## Abstract

**Background:**

Biomarkers of inflammation and adiponectin are associated with cardiovascular autonomic neuropathy (CAN) in cross-sectional studies, but prospective data are scarce. This study aimed to assess the associations of biomarkers of subclinical inflammation and adiponectin with subsequent changes in heart rate (HR) and heart rate variability (HRV) in non-diabetic and diabetic individuals.

**Methods:**

Data are based on up to 25,050 person-examinations for 8469 study participants of the Whitehall II cohort study. Measures of CAN included HR and several HRV indices. Associations between baseline serum levels of high-sensitivity C-reactive protein (hsCRP), interleukin (IL)-6, IL-1 receptor antagonist (IL-1Ra) and adiponectin and 5-year changes in HR and six HRV indices were estimated using mixed-effects models adjusting for age, sex, ethnicity, body mass index (BMI), metabolic covariates and medication. A modifying effect of diabetes was tested.

**Results:**

Higher levels of IL-1Ra were associated with higher increases in HR. Additional associations with measures of HRV were observed for hsCRP, IL-6 and IL-1Ra, but these associations were explained by BMI and other confounders. Associations between adiponectin, HR and HRV differed depending on diabetes status. Higher adiponectin levels were associated with more pronounced decreases in HR and increases in three measures of HRV reflecting both sympathetic and vagal activity, but these findings were limited to individuals with type 2 diabetes.

**Conclusions:**

Higher IL-1Ra levels appeared as novel risk marker for increases in HR. Higher adiponectin levels were associated with a more favourable development of cardiovascular autonomic function in individuals with type 2 diabetes independently of multiple confounders.

**Electronic supplementary material:**

The online version of this article (10.1186/s12933-017-0634-3) contains supplementary material, which is available to authorized users.

## Background

Cardiovascular autonomic neuropathy (CAN) is a frequent complication in patients with type 2 diabetes with prevalences reaching up to 60% after a disease duration of 15 years [1]. CAN is also observed in individuals with prediabetes and in older people, although in less severe forms [2] In addition to established cardiometabolic risk factors, CAN is an independent predictor of cardiovascular morbidity such as myocardial ischaemia and stroke, progression of diabetic nephropathy and overall mortality [3–5].

Symptoms of CAN are usually preceded by pathological changes in indices of resting heart rate (HR) and its variability (HRV) occurring early after the manifestation of diabetes, but also in early non-diabetic dysglyceamia [6]. Measurements of HRV are commonly used for the objective assessment of cardiac autonomic function in epidemiological studies and for the analysis of its association with novel risk factors in the early stages of CAN [7].

Risk factors for CAN are only partly understood. Old age, hyperglycaemia and further obesity-associated metabolic derangements contribute to the incidence of CAN, so that optimised glycaemic control and a multifactorial approach targeting cardiovascular risk factors using lifestyle-based and pharmacological strategies represent the cornerstone of current prevention guidelines [1] However, there are currently no therapeutic options aiming at other pathomechanisms of CAN. One of the mechanisms that has been implicated in the development of CAN is subclinical inflammation [7, 8]. Higher levels of proinflammatory biomarkers of inflammation such as high-sensitivity C-reactive protein (hsCRP) and interleukin (IL)-6 have been found associated with impaired HRV and higher odds of CAN [7]. Additionally, higher rather than lower circulating levels of the insulin-sensitising and potentially atheroprotective adipokine adiponectin [9] have been shown to be associated with CAN [10] or reduced HRV [11] in patients with type 2 diabetes.

Importantly, almost all available data on the relationship between subclinical inflammation and HRV or CAN are based on cross-sectional studies which does not allow for conclusions on causality, as the direction of associations may be bidirectional (reverse causality). Given the bidirectional interaction between the autonomic nervous system and inflammation [8] and the risk of reverse causality in the association between inflammation and cardiovascular phenotypes [12], large prospective studies are needed to elucidate the temporal relationships between biomarkers of inflammation including adiponectin and CAN.

Therefore, we aimed to investigate the prospective associations of baseline levels of biomarkers of subclinical inflammation [hsCRP, IL-6, IL-1 receptor antagonist (IL-1Ra)] and adiponectin with subsequent 5-year changes in HR and HRV in individuals with and without diabetes in large cohort.

## Methods

### Study participants, procedures and measurements

Participants are from the Whitehall II Study, an occupational cohort of 10,308 British civil servants (6896 men and 3412 women aged 35–55 years) of mainly white ethnicity recruited between 1985 and 1988 (phase 1) [13].

The cohort has been followed at eight subsequent phases. Every second phase (~ 5 years apart) included a clinical health examination (phases 1, 3, 5, 7, and 9). Data from phases 3, 5, 7, and 9 form the basis for this study as measurements of inflammation, HR, HRV and a standard 2-h 75 g OGTT were performed at those phases. Diabetes was diagnosed at screening by OGTT (WHO criteria) or by self-reported diagnosis. Participants fasting ≥ 8 h were classified as having diabetes if fasting- or 2-h plasma glucose value were ≥ 7.0 or 11.1 mmol/l respectively. Participants fasting < 8 h were classified with diabetes if any of their two glucose measurements were ≥ 11.1 mmol/l. Blood pressure and lipids were measured as described [13]. Patients with heart disease including ischemic heart disease and arrhythmias at baseline were excluded from analyses.

During follow-up, participants were censored if they died, were lost to follow-up or developed heart disease e.g. ischemic heart disease and arrhythmias either diagnosed at study examination or by register based follow-up, which comprised 1809 (6.0%) person-examinations.

Serum levels of hsCRP, IL-6, IL-1Ra and total adiponectin were measured as described [14, 15]. We excluded 543 (1.9%) person-examinations with hsCRP > 10 mg/l as indicator of acute infections.

Five-minute resting 12-lead ECG recordings were obtained subsequent to 5 min of supine rest. Recordings were filtered through an automated algorithm, allowing the analyses of suitable R–R intervals without the presence of arrhythmias, ectopic beats and branch blocks. Indices of HRV were analysed in the time domain [the standard deviation of all intervals between R waves with normal-to-normal conduction (SDNN) and the root mean square of the sum of the squares of differences between consecutive R–R intervals (RMSSD)] and by utilizing a Blackman-Tukey algorithm also in the frequency domain low-frequency (LF) power (in the 0.04–0.15 Hz frequency band), high-frequency (HF) power (in the 0.15–0.4 Hz frequency band) and total power (in the ≤ 0.4 Hz frequency band). The ratio between LF and HF power (LF/HF ratio) was calculated.

Information on smoking habits, physical activity and medication use were gathered by self-administered questionnaires [13].

### Statistical analysis

This analysis used HR and six HRV indices (SDNN, RMSSD, HF power, LF power, LF/HF ratio and total power) as outcomes. All except HR were log-transformed prior to analysis to fulfil the assumption of normally distributed model residuals.

The following biomarkers of subclinical inflammation were included as exposures: hsCRP, IL-6, IL-1Ra and adiponectin (all log_2_ transformed). Adiponectin and IL-1Ra were measured only in a case-cohort subsample nested within the Whitehall II study. [14, 15].

Up to 25,050 person-examinations for 8469 participants were analysed (9459 person-examinations for the 3421 participants in the subcohort). In each 5-year observation window of two consecutive phases, we studied the associations of baseline levels of biomarkers of subclinical inflammation and follow-up levels of the different outcomes, adjusting for baseline level of the outcome.

All analyses were adjusted for age, sex, ethnicity, study phase and diabetes (model 1). Additional adjustments included BMI and physical activity (model 2) and smoking, systolic blood pressure, total cholesterol, triglycerides, tricyclic antidepressants, diuretics and beta blockers (model 3). For all outcomes except HR, analyses were also adjusted for HR obtained as part of the HRV analyses. To compare the estimated associations across models 1–3 for a given outcome and exposure, we used a complete-case approach, limiting the analyses to data with complete information on all covariates in model 3. The same individual may contribute with more than one observation to the analyses. To account for the likely correlation of repeated measurements within the same participant, we used mixed-effects models with a random intercept and a random slope for time. Individuals changing diabetes state during the study contributed with person-examinations first to the non-diabetes group and later to the diabetes group.

Since prospective studies on adiponectin and cardiovascular outcomes have indicated that the direction of the association may depend on diabetes status (positive associations between adiponectin and cardiovascular events or mortality in individuals with diabetes rather than inverse associations in non-diabetic individuals) [16], we tested for a modifying effect of diabetes on the association between HR and HRV indices with adiponectin. As sensitivity analysis, we also calculated models including interaction terms with diabetes for the three biomarkers of inflammation (i.e. hsCRP, IL-6 and IL-1Ra). To assess if body composition estimated by waist circumference instead of BMI would affect model estimates, we repeated the analyses replacing BMI with waist circumference.

Heart rate and HRV indices are highly interrelated, and the same is true for the inflammation-related biomarkers and adiponectin. Therefore, correction for multiple testing would likely inflate the risk of type II errors and was therefore not applied.

Statistical analyses were performed in R version 3.3.1 (The R Foundation for Statistical Computing) and SAS version 9.2 (SAS Institute, Cary, NC).

## Results

The study population included 7769 participants at the first study phase and 5427 individuals at the last study phase. Median time between two subsequent phases was 5.4 years (25th; 75th percentiles 5.0; 5.8 years). The prevalence of diabetes increased from 3.0 to 12.8%. Serum levels of hsCRP and IL-6 increased during follow-up, while levels of IL-1Ra and adiponectin remained stable. All indices of HRV decreased over the study period (Table 1).

**Table 1:** Characteristics of the study population at each study phase

	Phase 3 (1991–1993)	Phase 5 (1997–1999)	Phase 7 (2002–2004)	Phase 9 (2007–2009)
N	7769	6105	5749	5427
Men (%)	69.4 (68.3; 70.4)	70.9 (69.8; 72.1)	70.5 (69.3; 71.6)	70.0 (68.8; 71.2)
White ethnicity (%)	90.3 (89.6; 91.0)	91.8 (91.1; 92.5)	92.1 (91.3; 92.8)	91.9 (91.2; 92.6)
Age (years)	50.0 (6.0)	55.6 (6.0)	60.8 (5.9)	65.5 (5.8)
Height (cm)	171.9 (9.5)	172.1 (9.2)	171.0 (9.3)	170.7 (9.3)
BMI (kg/m^2^)	25.3 (3.7)	26.1 (3.9)	26.5 (4.2)	26.7 (4.4)
Waist circumference (cm)	85.7 (11.5)	90.5 (11.7)	93.1 (12.1)	94.5 (12.0)
Current smokers (%)	13.0 (12.3; 13.8)	9.5 (8.8; 10.3)	7.9 (7.2; 8.6)	5.5 (4.9; 6.1)
Moderate to vigorous exercise (hours/week)	2.0 (1.0; 5.0)	11.8 (4.8; 20.3)	11.5 (4.3; 20.0)	–
Alcohol intake (units/week)	6.0 (2.0; 14.0)	9.0 (3.0; 20.0)	8.0 (3.0; 17.0)	7.0 (2.0; 15.0)
Diabetes (%)	3.0 (2.6; 3.4)	5.6 (5.1; 6.2)	8.9 (8.1; 9.6)	12.8 (11.9; 13.7)
Medication				
Antihypertensive treatment (%)	6.7 (6.2; 7.3)	11.5 (10.7; 12.3)	21.2 (20.1; 22.3)	32.7 (31.4; 34.0)
Lipid-lowering treatment (%)	0.7 (0.5; 0.9)	2.5 (2.1; 2.9)	9.2 (8.5; 10.0)	29.9 (28.7; 31.1)
Tricyclic antidepressants (%)^a^	1.9 (1.6; 2.2)	2.7 (2.3; 3.1)	2.9 (2.5; 3.4)	3.5 (3.0; 4.0)
Diuretics (%)	6.1 (5.6; 6.7)	3.0 (2.6; 3.5)	7.6 (7.0; 8.4)	10.8 (10.0; 11.7)
Beta blockers (%)	0.6 (0.4; 0.8)	4.7 (4.2; 5.3)	8.0 (7.3; 8.7)	7.4 (6.7; 8.1)
Blood measurements				
Total cholesterol (mmol/l)	6.5 (1.2)	5.9 (1.1)	5.8 (1.0)	5.3 (1.1)
HDL cholesterol (mmol(l)	1.4 (0.4)	1.5 (0.4)	1.6 (0.4)	1.6 (0.5)
LDL cholesterol (mmol/l)	4.4 (1.0)	3.9 (0.9)	3.6 (0.9)	3.1 (1.0)
Triglycerides (mmol/l)	1.5 (1.1)	1.4 (0.9)	1.4 (0.9)	1.3 (0.7)
Systolic blood pressure (mmHg)	120.6 (13.6)	123.0 (16.5)	128 (16.8)	125.7 (16.1)
Diastolic blood pressure (mmHg)	79.7 (9.4)	77.5 (10.6)	74.4 (10.4)	71.3 (10.2)
CRP (mg/dl)	0.9 (0.4; 1.8)	1.0 (0.5; 2.0)	1.2 (0.6; 2.4)	–
IL-6 (pg/ml)	1.4 (1.0; 2.0)	1.4 (1.0; 2.0)	1.7 (1.3; 2.4)	–
IL-1Ra (pg/ml)^b^	251 (195; 329)	340 (266; 437)	347 (271; 458)	347 (273; 450)
Adiponectin (µg/ml)^b^	8.5 (6.2; 12.1)	8.5 (6.2; 12.1)	8.3 (5.9; 11.9)	8.4 (5.8; 12.9)
Heart rate measurements				
Heart rate from ECG (bpm)	64.7 (10.7)	67.3 (11.2)	67.9 (11.6)	66.5 (11.5)
SDNN (ms)	–	34.2 (26.0; 44.8)	33.7(25.4; 44.7)	29.8 (22.0; 40.1)
RMSSD (ms)	–	20.3 (13.6; 29.4)	20.5(13.6; 30.2)	17.5 (11.8; 26.6)
Low-frequency power (ms^2^)	–	309 (1657; 569)	284 (157; 524)	222 (113; 439)
High-frequency power (ms^2^)	–	128 (615; 251)	115(56; 236)	86 (42; 186)
LF/HF ratio	–	2.6 (1.5; 4.1)	2.6 (1.5; 4.0)	2.6 (1.6; 4.1)
Total power (ms^2^)	–	1048 (601;1784)	1004 (568; 1749)	779 (423; 1411)

Data are means (SD), medians (25th; 75th percentiles) or proportions (95% CI)

^a^At phase 3, we are not able to separate tricyclic antidepressants from other antidepressants

^b^Subsample (n = 9459 records)

For hsCRP, IL-6 and IL-1Ra, we found no modifying effect of diabetes on any of the associations, therefore results are presented for the whole study sample combined, adjusting for diabetes (Table 2). Higher baseline levels of IL-1Ra were associated with larger increases in HR at all adjustment levels. Conversely, higher IL-1Ra was associated with a decrease in SDNN, LF power and total power in model 1, but effects were attenuated after further adjustment for BMI and physical activity. Higher levels of hsCRP were associated with more pronounced increases in HR, and both hsCRP and IL-6 were associated with larger decreases in the LF/HF ratio, but again these associations were partially mediated by BMI and not significant in subsequent models (Additional file 1: Table S1).

**Table 2:** Effects (with 95% CI) of a doubling in the inflammatory marker at baseline on 5-year changes in heart rate and HRV indices

Outcome	Model	hsCRP			IL-6			IL-1Ra		
		n	Estimate	p	n	Estimate	p	n	Estimate	p
Heart rate (bpm)	1	13,251	0.2 (0.1; 0.3)	< 0.001	12,971	0.1 (0.0; 0.3)	0.100	7241	0.7 (0.4; 1.0)	< 0.001
	2	13,251	0.1 (0.0; 0.2)	0.052	12,971	0.0 (− 0.2; 0.2)	0.910	7241	0.6 (0.3; 1.0)	< 0.001
	3	13,251	0.1 (0.0; 0.2)	0.108	12,971	0.0 (− 0.2; 0.1)	0.696	7241	0.6 (0.2; 0.9)	0.002
SDNN (% diff)	1	4258	0.1 (− 0.7; 0.9)	0.796	4286	− 0.3 (− 1.8; 1.3)	0.731	2399	− 3.2 (− 5.7; − 0.6)	0.018
	2	4258	0.3 (− 0.6; 1.2)	0.502	4286	− 0.1 (− 1.6; 1.6)	0.949	2399	− 2.3 (− 5.0; 0.6)	0.120
	3	4258	0.5 (− 0.4; 1.4)	0.267	4286	0.3 (− 1.3; 1.9)	0.750	2399	− 1.8 (− 4.6; 1.1)	0.225
RMSSD (% diff)	1	4258	0.5 (− 0.6; 1.7)	0.376	4286	0.7 (− 1.5; 2.8)	0.546	2399	− 2.0 (− 5.6; 1.7)	0.277
	2	4258	0.6 (− 0.7; 1.9)	0.360	4286	0.8 (− 1.5; 3.0)	0.513	2399	− 1.1 (− 4.9; 2.9)	0.580
	3	4258	0.6 (− 0.7; 1.9)	0.354	4286	0.7 (− 1.5; 3.0)	0.529	2399	− 0.9 (− 4.9; 3.1)	0.644
Low frequency power (% diff)	1	4258	− 0.9 (− 2.7; 0.9)	0.342	4286	− 1.8 (− 5.1; 1.6)	0.299	2399	− 7.1 (− 12.5; − 1.4)	0.016
	2	4258	− 0.1 (− 2.1; 1.9)	0.930	4286	− 0.7 (− 4.2; 2.9)	0.694	2399	− 3.9 (− 9.8; 2.5)	0.227
	3	4258	0.5 (− 1.5; 2.5)	0.645	4286	0.1 (− 3.4; 3.8)	0.937	2399	− 2.7 (− 8.8; 3.9)	0.420
High frequency power (% diff)	1	4258	0.6 (− 1.5; 2.7)	0.588	4286	1.3 (− 2.5; 5.2)	0.510	2399	− 5.2 (− 11.3; 1.3)	0.114
	2	4258	1.1 (− 1.2; 3.4)	0.347	4286	2.0 (− 2.0; 6.1)	0.334	2399	− 2.8 (− 9.5; 4.3)	0.430
	3	4258	1.2 (− 1.1; 3.5)	0.299	4286	2.1 (− 1.9; 6.3)	0.312	2399	− 2.3 (− 9.1; 5.0)	0.524
LF/HF ratio (% diff)	1	4258	− 1.4 (− 2.7; − 0.2)	0.027	4286	− 3.0 (− 5.3; − 0.6)	0.013	2399	− 1.7 (− 5.8; 2.6)	0.433
	2	4258	− 1.2 (− 2.6; 0.2)	0.099	4286	− 2.6 (− 5.0; − 0.1)	0.039	2399	− 1.0 (− 5.4; 3.7)	0.684
	3	4258	− 0.7 (− 2.1; 0.7)	0.314	4286	− 1.8 (− 4.3; 0.7)	0.152	2399	− 0.3 (− 4.8; 4.5)	0.913
Total power (% diff)	1	4258	0.0 (− 1.6; 1.6)	0.973	4286	− 0.7 (− 3.7; 2.3)	0.630	2399	− 6.4 (− 11.3; − 1.3)	0.015
	2	4258	0.5 (− 1.3; 2.3)	0.596	4286	− 0.1 (− 3.3; 3.1)	0.939	2399	− 4.2 (− 9.5; 1.4)	0.142
	3	4258	1.0 (− 0.8; 2.8)	0.282	4286	0.7 (− 2.5; 3.9)	0.690	2399	− 3.2 (− 8.7; 2.6)	0.274

*bpm* beats per minute, *diff*. difference, *N* number of person-examinations used in the particular analysis

p: *p* value for the test of the effect being equal to zero

Model 1: Adjusted for age, sex, ethnicity, study phase and diabetes. For HRV indices, further adjustment for heart rate obtained as part of the HRV analyses

Model 2: Further adjustment for BMI and physical activity

Model 3: Further adjustment for smoking, systolic blood pressure, total cholesterol, triglycerides, tricyclic antidepressants, diuretics and beta blockers

In contrast, we found a modifying effect of diabetes on the association between serum levels of total adiponectin and future changes in HR and several HRV indices. In participants with type 2 diabetes, higher levels of total adiponectin were associated with a decrease in HR and increases in values of SDNN and RMSSD (time-domain indices) and of the LF power (frequency-domain index) (Table 3). These association were not only significant after initial adjustment for age, sex, ethnicity, diabetes, HR and study phase, but remained largely unaffected by further adjustment for BMI, lifestyle factors, blood pressure, serum lipids and medication (tricyclic antidepressants, diuretics and beta blockers). In contrast, non-diabetic individuals only showed an inverse association between adiponectin and HR in model 1 (Table 3).

**Table 3:** Effects (with 95% CI) of a doubling in adiponectin at baseline on 5-year changes in heart rate and HRV indices by diabetes status

	Model	No diabetes			Diabetes			p_interaction_
		n	Estimate	p	n	Estimate	p	
Heart rate (bpm)	1	6841	*−* *0.3* (*−* *0.6; 0.0*)	*0.029*	400	*−* *1.5* (*−* *2.5*; *−* *0.5*)	*0.003*	*0.021*
	2	6841	− 0.2 (− 0.5; 0.1)	0.119	400	*−* *1.4* (*−* *2.4*; *−* *0.4*)	*0.005*	*0.022*
	3	6841	− 0.2 (− 0.5; 0.2)	0.313	400	*−* *1.3* (*−* *2.3*; *−* *0.3*)	*0.014*	*0.035*
SDNN (% diff.)	1	2210	1.3 (− 1.1; 3.7)	0.287	189	*10.1* (*2.5*; *18.2*)	*0.009*	*0.027*
	2	2210	0.7 (− 1.7; 3.1)	0.591	189	*9.3* (*1.8*; *17.4*)	*0.015*	*0.028*
	3	2210	0.0 (− 2.4; 2.5)	0.999	189	*8.2* (*0.7*; *16.3*)	*0.032*	*0.036*
RMSSD (% diff.)	1	2210	0.3 (− 2.9; 3.5)	0.877	189	*13.3* (*2.7*; *25.0*)	*0.013*	*0.018*
	2	2210	− 0.3 (− 3.6; 3.0)	0.844	189	*12.5* (*2.0*; *24.1*)	*0.019*	*0.018*
	3	2210	− 0.7 (− 4.0; 2.7)	0.680	189	*11.8* (*1.2*; *23.4*)	*0.027*	*0.022*
Low frequency power (% diff.)	1	2210	3.2 (− 2.1; 8.7)	0.245	189	*27.5* (*8.7*; *49.5*)	*0.003*	*0.011*
	2	2210	1.2 (− 4.1; 6.8)	0.660	189	*24.9* (*6.5*; *46.5*)	*0.006*	*0.012*
	3	2210	− 0.3 (− 5.7; 5.4)	0.916	189	*22.5* (*4.3*; *43.9*)	*0.013*	*0.014*
All participants								
High frequency power (% diff.)	1	2399	1.7 (− 3.8; 7.6)	0.552				0.267
	2	2399	0.1 (− 5.4; 6.1)	0.960				0.303
	3	2399	− 1.2 (− 6.9; 4.9)	0.696				0.119
LF/HF ratio (% diff.)	1	2399	3.2 (− 0.5; 7.0)	0.090				0.319
	2	2399	2.9 (− 0.8; 6.8)	0.130				0.314
	3	2399	2.7 (− 1.2; 6.8)	0.171				0.342
Total power (% diff.)	1	2399	4.1 (− 0.5; 9.0)	0.081				0.065
	2	2399	2.6 (− 2.0; 7.5)	0.274				0.067
	3	2399	1.1 (− 3.7; 6.1)	0.662				0.086

For heart rate 400 out of 7241 records were for participants with diabetes. For the HRV indices, 189 out of 2399 records were for participants with diabetes

*bpm* beats per minute, *diff*. difference, *n* number of person-examinations used in the particular analysis

p: p-value for the test of the effect being equal to zero

p_interaction_: p-value for interaction with diabetes status

Model 1: Adjusted for age, sex, ethnicity, study phase and diabetes. For HRV indices, further adjustment for heart rate obtained as part of the HRV analyses

Model 2: Further adjustment for BMI and physical activity

Model 3: Further adjustment for smoking, systolic blood pressure, total cholesterol, triglycerides, tricyclic antidepressants, diuretics and beta blockers

Adjusting for waist circumference instead of BMI did not affect model estimates (Additional file 1: Table S1 and Additional file 2: Table S2).

## Discussion

This study has two major findings: (i) Higher baseline levels of hsCRP, IL-6 and IL-1Ra showed associations with larger increases in HR and decreases in several HRV indices after initial adjustment for age, sex and diabetes status. BMI represented a substantial confounder for all associations expect for the one between higher IL-1Ra levels and larger increases in HR, which remained significant in the fully adjusted model. (ii) Higher baseline adiponectin levels were association with smaller increases in HR and larger increases in HRV indices reflecting both sympathetic and vagal activity. However, these associations were present only in individuals with type 2 diabetes, but absent in the non-diabetic study population.

### Biomarkers of subclinical inflammation (hsCRP, IL-6, IL-1Ra), HR and HRV

Our data on biomarkers of subclinical inflammation, HR and HRV are novel, as only two small prospective studies that investigated the temporal relationship between hsCRP and vagal tone assessed by HRV measurement of HF power [17, 18].

We show that higher IL-1Ra levels were associated with more pronounced increases in resting HR after adjustment for a range of potentially confounding factors. Given the established link between higher resting HR and increased risk of cardiovascular outcomes (e.g. coronary heart disease, sudden cardiac death, stroke, atrial fibrillation) and mortality [19], this finding implicates cytokines of the IL-1 family in the regulation of HR and in cardiovascular risk. As antagonist of the proinflammatory cytokine IL-1β, IL-1Ra has no agonist activity of its own and is thus unlikely to impair cardiac function [20]. Higher IL-1Ra levels are related to a higher risk of cardiovascular events in population-based studies [21] and have been proposed to reflect the presence of metabolic and/or inflammation-related cardiovascular risk factors that may involve the activation of the NLR family pyrin domain containing 3 (NLRP3) inflammasome and increased IL-1β activity [21, 22]. Higher IL-1Ra could thus be interpreted as result of an endogenous but eventually insufficient counter-regulation.

For the three biomarkers of subclinical inflammation selected in this study, we found five significant associations with changes in various HRV indices (LF power, LF/HF ratio and total power) out of 18 associations tested. Therefore, our study provides some evidence that subclinical inflammation may precede both impaired sympathetic and vagal activity, but at the same time indicates that BMI represents a major confounder in this context. Overall, effect sizes were small and no clear pattern of associations emerged, so that we did not find a pronounced impact of baseline levels of these biomarkers on impairments in HRV in our population-based study population. Therefore, our results differ from associations between inflammation-related biomarkers and cardiac autonomic dysfunction in cross-sectional studies [7]. Moreover, we could not confirm the association between CRP levels and increases in HF power from a small prospective study [18]. Although direct effects of immune activation e.g. induced by lipopolysaccharide on HR and HRV have been demonstrated, it is important to emphasise that these studies involve acute elevations of proinflammatory cytokines that exceed circulating levels in the general population by one to two orders of magnitude [23]. Collectively, these findings raise the question whether associations between biomarkers of inflammation and HRV in cross-sectional studies are mainly caused by effects of the autonomic nervous system on inflammatory pathways [8]. Another small prospective study indeed showed an inverse association between HF–HRV and CRP 4 years later [17]. It is also possible that subclinical inflammation is so closely linked to the autonomic regulation that changes in inflammation are associated with more immediate changes in HRV. This would explain that no temporal association to future HRV was seen when adjusting for baseline HRV in the present study.

### Adiponectin, HR and HRV

The present study demonstrates that higher serum levels of total adiponectin were associated with beneficial changes in HR and several measures of HRV 5 years after blood sampling in patients with diabetes. The associations were present for three of the six HRV indices, SDNN, RMSSD and LF power. RMSSD is a marker of parasympathetic autonomic tone whereas SDNN and LF power are mainly sympathetic measures. This suggests that in diabetes patients adiponectin could have neuroprotective effects on both branches of the autonomic nervous system. There were no significant associations between adiponectin and changes in HR or HRV in non-diabetic individuals indicating that adiponectin may not be associated with autonomic regulation in healthy people. Reduced HRV is not only strongly associated with diabetes in the general population, but already found in youth with type 2 diabetes and in adults with prediabetes or insulin resistance [24–27]. However, further research is necessary to elucidate to what extent determinants of reduced HRV and CAN such as adiponectin and other biomarkers may depend on age and on the degree of glucose intolerance and hyperglycaemia.

We are not aware of previous prospective studies that assessed the potential link between adiponectin levels and future changes in HR or HRV. Three cross-sectional studies reported that higher adiponectin levels were associated with a reduced LF/HF ratio [28], reduced very-low-frequency (VLF) power [11] and higher odds of CAN [10] in individuals with type 2 diabetes, whereas a study based on an occupational sample (prevalence of diabetes 2.3%) observed no associations between adiponectin and HRV indices [29]. Thus, the direction of associations between adiponectin and HRV or CAN differs between these aforementioned cross-sectional studies and our prospective study. Associations between higher adiponectin levels and higher cardiovascular risk have been reported before in individuals with type 2 diabetes [16] and may be explained as counterregulatory phenomenon as discussed above for IL-1Ra [30] The second explanation is of interest because adiponectin is upregulated by natriuretic peptides which are direct markers of coronary heart disease and could mediate such associations [31].

Our findings of favourable associations between baseline adiponectin levels and changes in both HRV and indices of HRV is in line with the inverse association between higher adiponectin levels and lower incidence of distal sensorimotor polyneuropathy in an older population-based cohort with more than half of the participants having prediabetes or type 2 diabetes [32]. However, the latter association was mediated by obesity and other confounders, whereas the association with measures of early CAN in the present study was robust to extensive adjustment.

### Strengths and limitations

Strengths of this study are the large sample size, the comprehensive measurements of outcomes and exposures assessed simultaneously, the extensive adjustment for confounders, the stratification by diabetes status and the exclusion of individuals with elevated hsCRP to reduce the impact of acute inflammatory states. Importantly, the prospective design allowed us to assess the temporal association between inflammation and HRV changes, whereas associations in cross-sectional studies may be attributable to reverse causality, i.e. impact of HR and HRV on biomarkers of inflammation. Limitations include the observational design that precludes causal inferences and the selection of four pro- and anti-inflammatory biomarkers whereas others (e.g. IL-18) also merit further research. Furthermore, the study population was predominantly of European descent, so that data cannot be generalised to other ethnic groups. Also, the cohort is based on people employed as civil servants at the study start excluding e.g. unemployed people and people employed in the private sector. The study may therefore not be fully generalizable to the general population.

## Conclusions

Taken together, we observed that higher IL-1Ra levels preceded larger increases in resting HR. Further analyses of hsCRP, IL-6 and IL-1Ra pointed towards BMI-dependent inverse associations with indices of HRV. Higher adiponectin levels showed associations with less pronounced increases in HR and a more favourable development of sympathetic and vagal tone in individuals with type 2 diabetes. The latter associations were absent in non-diabetic individuals, so that further studies are required to clarify in which contexts adiponectin may have therapeutically interesting properties for the long-term development of autonomic regulation.

## Additional files

**Additional file 1: Table S1.** Effects (with 95% CI) of a doubling in the inflammatory marker at baseline on 5-year changes in heart rate and HRV indices (adjusting for waist cirumference).

**Additional file 2: Table S2.** Effects (with 95% CI) of a doubling in adiponectin at baseline on 5-year changes in heart rate and HRV indices by diabetes status (adjusting for waist cirumference).

## Abbreviations

CAN: cardiovascular autonomic neuropathy
HF power: high-frequency power
HR: heart rate
HRV: heart rate variability
HsCRP: high-sensitivity C-reactive protein
IL-6: interleukin 6
IL-1Ra: interleukin 1 receptor antagonist
LF power: low-frequency power
LF/HF ratio: low frequency/high frequency power ratio
RMSSD: the root mean square of the sum of the squares of differences between consecutive R–R intervals
SDNN: the standard deviation of all intervals between R waves with normal-to-normal conduction
