# Supplementary material for: Adiponectin, biomarkers of inflammation and changes in cardiac autonomic function: Whitehall II study
Source: Cardiovasc Diabetol. 2017 Dec 1;16:153. doi: 10.1186/s12933-017-0634-3 (PMC5710029; doi:10.1186/s12933-017-0634-3)
Supplement: Supplementary file 1 — Additional file 1: Table S1. Effects (with 95% CI) of a doubling in the inflammatory marker at baseline on 5-year changes in heart rate and HRV indices (adjusting for waist cirumference). [file 12933_2017_634_MOESM1_ESM.docx]

Table S1 Effects (with 95% CI) of a doubling in the inflammatory marker at baseline on 5-year changes in heart rate and HRV indices (adjusting for waist circumference)

|  |  | hsCRP |  |  | IL-6 |  |  | IL-1Ra |  |  |
| --- | --- | --- | --- | --- | --- | --- | --- | --- | --- | --- |
| Outcome | Model | n | Estimate | p | n | Estimate | p | n | Estimate | p |
| Heart rate (bpm) | 1 | 12687 | **0.2 (0.1;0.3)** | **<0.001** | 12408 | **0.2 (0.0;0.4)** | **0.048** | 7213 | **0.7 (0.4;1.1)** | **<0.001** |
|  | 2 | 12687 | 0.1 (0.0;0.2) | 0.164 | 12408 | 0.0 (-0.2;0.2) | 0.952 | 7213 | **0.5 (0.1;0.8)** | **0.006** |
|  | 3 | 12687 | 0.1 (0.0;0.2) | 0.263 | 12408 | -0.1 (-0.2;0.1) | 0.601 | 7213 | **0.4 (0.1;0.8)** | **0.021** |
| SDNN (% diff) | 1 | 4159 | 0.2 (-0.6;1.1) | 0.618 | 4187 | -0.3 (-1.8;1.3) | 0.723 | 2392 | **-3.2 (-5.8;-0.6)** | **0.017** |
|  | 2 | 4159 | 0.3 (-0.6;1.2) | 0.534 | 4187 | -0.3 (-1.9;1.4) | 0.764 | 2392 | -2.4 (-5.1;0.5) | 0.106 |
|  | 3 | 4159 | 0.5 (-0.4;1.4) | 0.287 | 4187 | 0.0 (-1.6;1.7) | 0.959 | 2392 | -1.9 (-4.7;1.1) | 0.206 |
| RMSSD (% diff) | 1 | 4159 | 0.7 (-0.5;1.9) | 0.244 | 4187 | 0.8 (-1.4;3.0) | 0.479 | 2392 | -2.1 (-5.6;1.6) | 0.264 |
|  | 2 | 4159 | 0.7 (-0.6;2.0) | 0.287 | 4187 | 0.8 (-1.5;3.1) | 0.508 | 2392 | -1.1 (-4.9;2.9) | 0.599 |
|  | 3 | 4159 | 0.7 (-0.6;2.0) | 0.286 | 4187 | 0.7 (-1.6;3.1) | 0.541 | 2392 | -0.9 (-4.8;3.2) | 0.664 |
| Low frequency power (% diff) | 1 | 4159 | -0.7 (-2.6;1.1) | 0.434 | 4187 | -1.8 (-5.1;1.7) | 0.317 | 2392 | **-7.0 (-12.5;-1.3)** | **0.017** |
|  | 2 | 4159 | -0.3 (-2.3;1.8) | 0.776 | 4187 | -1.2 (-4.7;2.5) | 0.535 | 2392 | -4.3 (-10.3;2.1) | 0.183 |
|  | 3 | 4159 | 0.3 (-1.8;2.3) | 0.807 | 4187 | -0.3 (-4.0;3.4) | 0.852 | 2392 | -3.1 (-9.3;3.5) | 0.349 |
| High frequency power (% diff) | 1 | 4159 | 0.9 (-1.2;3.0) | 0.392 | 4187 | 1.5 (-2.4;5.5) | 0.462 | 2392 | -5.3 (-11.4;1.2) | 0.108 |
|  | 2 | 4159 | 1.3 (-0.9;3.6) | 0.252 | 4187 | 2.0 (-2.1;6.2) | 0.345 | 2392 | -2.9 (-9.5;4.3) | 0.425 |
|  | 3 | 4159 | 1.4 (-0.9;3.8) | 0.220 | 4187 | 2.0 (-2.1;6.3) | 0.339 | 2392 | -2.4 (-9.2;5.0) | 0.514 |
| LF/HF ratio (% diff) | 1 | 4159 | **-1.7 (-2.9;-0.4)** | **0.012** | 4187 | **-3.1 (-5.5;-0.8)** | **0.010** | 2392 | -1.5 (-5.7;2.8) | 0.485 |
|  | 2 | 4159 | **-1.7 (-3.0;-0.2)** | **0.022** | 4187 | **-3.1 (-5.5;-0.5)** | **0.017** | 2392 | -1.3 (-5.8;3.4) | 0.575 |
|  | 3 | 4159 | -1.2 (-2.6;0.2) | 0.099 | 4187 | -2.3 (-4.8;0.3) | 0.079 | 2392 | -0.6 (-5.2;4.2) | 0.793 |
| Total power (% diff) | 1 | 4159 | 0.2 (-1.5;1.9) | 0.828 | 4187 | -0.8 (-3.8;2.4) | 0.626 | 2392 | **-6.5 (-11.4;-1.3)** | **0.014** |
|  | 2 | 4159 | 0.4 (-1.4;2.2) | 0.680 | 4187 | -0.6 (-3.8;2.7) | 0.711 | 2392 | -4.7 (-10.0;1.0) | 0.103 |
|  | 3 | 4159 | 0.9 (-0.9;2.8) | 0.342 | 4187 | 0.1 (-3.1;3.5) | 0.947 | 2392 | -3.6 (-9.1;2.2) | 0.219 |

bpm, beats per minute; diff., difference; N, number of person-examinations used in the particular analysis.

p: p-value for the test of the effect being equal to zero.

Model 1: Adjusted for age, sex, ethnicity, study phase and diabetes. For HRV indices, further adjustment for heart rate obtained as part of the HRV analyses.

Model 2: Further adjustment for waist circumference and physical activity.

Model 3: Further adjustment for smoking, systolic blood pressure, total cholesterol, triglycerides, tricyclic antidepressants, diuretics and beta blockers
