# Supplementary material for: Adiponectin, biomarkers of inflammation and changes in cardiac autonomic function: Whitehall II study
Source: Cardiovasc Diabetol. 2017 Dec 1;16:153. doi: 10.1186/s12933-017-0634-3 (PMC5710029; doi:10.1186/s12933-017-0634-3)
Supplement: Supplementary file 2 — Additional file 2: Table S2. Effects (with 95% CI) of a doubling in adiponectin at baseline on 5-year changes in heart rate and HRV indices by diabetes status (adjusting for waist cirumference). [file 12933_2017_634_MOESM2_ESM.docx]

Table S2 Effects (with 95% CI) of a doubling in adiponectin at baseline on 5-year changes in heart rate and HRV indices by diabetes status (adjusting for waist circumference)

|  |  | **No diabetes** |  |  | **Diabetes** |  |  |
| --- | --- | --- | --- | --- | --- | --- | --- |
|  | **Model** | **n** | **Estimate** | **p** | **n** | **Estimate** | **p** |
| Heart rate (bpm) | 1 | 6813 | **-0.3 (-0.6;0.0)** | **0.030** | 400 | **-1.5 (-2.5;-0.5)** | **0.002** |
|  | 2 | 6813 | -0.2 (-0.5;0.1) | 0.318 | 400 | **-1.4 (-2.4;-0.4)** | **0.006** |
|  | 3 | 6813 | -0.1 (-0.4;0.2) | 0.578 | 400 | **-1.2 (-2.3;-0.2)** | **0.015** |
| SDNN (% diff.) | 1 | 2204 | 1.3 (-1.0;3.7) | 0.273 | 188 | **9.1 (1.6;17.2)** | **0.016** |
|  | 2 | 2204 | 0.7 (-1.7;3.1) | 0.579 | 188 | **8.6 (1.2;16.6)** | **0.023** |
|  | 3 | 2204 | 0.0 (-2.5;2.6) | 0.991 | 188 | **7.4 (0.0;15.4)** | **0.050** |
| RMSSD (% diff.) | 1 | 2204 | 0.3 (-2.9;3.6) | 0.868 | 188 | **12.2 (1.8;23.8)** | **0.021** |
|  | 2 | 2204 | -0.4 (-3.7;2.9) | 0.803 | 188 | **11.6 (1.2;23.1)** | **0.027** |
|  | 3 | 2204 | -0.8 (-4.2;2.7) | 0.647 | 188 | **10.9 (0.5;22.4)** | **0.040** |
| Low frequency power (% diff.) | 1 | 2204 | 3.1 (-2.2;8.7) | 0.255 | 188 | **24.8 (6.4;46.3)** | **0.006** |
|  | 2 | 2204 | 1.2 (-4.1;6.8) | 0.653 | 188 | **23.3 (5.2;44.6)** | **0.010** |
|  | 3 | 2204 | -0.3 (-5.7;5.5) | 0.926 | 188 | **20.6 (2.7;41.6)** | **0.022** |
|  |  | **All** | **participants** |  |  |  |  |
|  |  | **n** | **Estimate** | **p** |  |  |  |
| High frequency power (% diff.) | 1 | 2392 | 1.7 (-3.9;7.6) | 0.555 |  |  |  |
|  | 2 | 2392 | 0.1 (-5.5;6.0) | 0.982 |  |  |  |
|  | 3 | 2392 | -1.3 (-7.0;4.8) | 0.676 |  |  |  |
| LF/HF ratio (% diff.) | 1 | 2392 | 3.0 (-0.7;6.9) | 0.108 |  |  |  |
|  | 2 | 2392 | 3.0 (-0.8;6.9) | 0.119 |  |  |  |
|  | 3 | 2392 | 2.8 (-1.1;6.9) | 0.161 |  |  |  |
| Total power (% diff.) | 1 | 2392 | 4.1 (-0.6;8.9) | 0.085 |  |  |  |
|  | 2 | 2392 | 2.8 (-1.9;7.7) | 0.252 |  |  |  |
|  | 3 | 2392 | 1.1 (-3.7;6.2) | 0.648 |  |  |  |

bpm, beats per minute; diff., difference; N, number of person-examinations used in the particular analysis; n, number of records.

p: p-value for the test of the effect being equal to zero.

p_interaction_: p-value for interaction with diabetes status

Model 1: Adjusted for age, sex, ethnicity, study phase and diabetes. For HRV indices, further adjustment for heart rate obtained as part of the HRV analyses.

Model 2: Further adjustment for waist circumference and physical activity.

Model 3: Further adjustment for smoking, systolic blood pressure, total cholesterol, triglycerides, tricyclic antidepressants, diuretics and beta blockers
